# A multi-view autoencoder architecture with self-adaptive feature recalibration and confidence-aware ensemble for heart disease classification

**DOI:** 10.1038/s41598-026-53138-7

**Published:** 2026-06-10

**Authors:** Abulfadhel Amer Saihood Altufaili, Dunya Mohammed Shleej

**Affiliations:** 1 Department of Electronic Technologies and Communications, Najaf Technical Institute, Al- Furat Al-Awsat Technical University, Najaf, Iraq; 2 Department of Medical Instrumentation Techniques, University of Imam Jaafer Sadiq, Najaf, Iraq

**Keywords:** Heart disease prediction, Multi-view autoencoder, Self-adaptive feature recalibration, Dynamic ensemble learning, Deep feature representation, Machine learning in healthcare, Clinical decision support systems, Computational biology and bioinformatics, Diseases, Health care, Mathematics and computing

## Abstract

Heart disease continues to pose a major challenge to global health, underscoring the need for early, accurate prediction models. In this study, we introduce a new hybrid intelligent framework designed to significantly improve heart disease classification. Our approach combines multi-view deep feature extraction, self-adaptive feature recalibration, and dynamic ensemble learning to deliver more reliable predictions. The process begins with a multi-view autoencoder that separately captures latent features from demographic, clinical, and diagnostic data. This separation preserves the unique information each data type offers, leading to richer and more meaningful feature representations. Next, we apply a self-adaptive feature recalibration mechanism that assigns importance weights to each feature based on the data itself. This ensures that features with stronger clinical relevance play a greater role in the model’s decision-making. Finally, we integrate a confidence-aware ensemble of three powerful classifiers—Extra Trees, Random Forest, and XGBoost. This ensemble dynamically adjusts the influence of each model depending on how confident they are at the instance level. We tested the proposed framework across five well-known heart disease datasets, using 10-fold cross-validation to ensure robustness. The results are promising: the model achieved an accuracy of 92.45%, sensitivity of 93.2%, specificity of 91.4%, and an F1-score of 91.4%. It consistently outperformed traditional machine learning methods, recent hybrid ensembles, and even state-of-the-art deep learning models like TabNet, SAINT, NODE, and TabTransformer. Statistical significance was confirmed via Friedman and Wilcoxon signed-rank tests (*p* < 0.001). To support interpretability, we used SHAP analysis, which highlighted key medical predictors such as chest pain type, number of major vessels, and ST depression. In summary, our results demonstrate that combining multi-view representation learning with self-adaptive feature recalibration and dynamic ensemble strategies leads to a highly effective, interpretable, and clinically relevant tool for early heart disease prediction. This framework holds strong promise for integration into smart clinical decision support systems, with future research aimed at validating it on larger and more diverse patient populations. Not applicable. This research does not involve a clinical trial or any prospective human experimentation.

## Introduction

Heart disease is one of the most serious global health threats, taking millions of lives each year (Di et al., 2024^1^;. That’s why early detection is essential to reducing death rates^2^. When caught early, the disease can often be managed or slowed down with the right treatment^3^. Unfortunately, diagnosing heart disease in time remains a challenge, especially in regions with limited access to healthcare^4^. These issues aren’t confined to developing countries—they’re also present in underserved communities within wealthier nations, making heart disease a truly widespread problem.

To address these challenges, machine learning (ML) has emerged as a powerful tool. With the ability to analyze massive amounts of data, ML can identify patterns and predict diseases like heart conditions with impressive accuracy. By using advanced algorithms, healthcare professionals can spot warning signs earlier and offer timely care—potentially saving lives^5^.

AI techniques have also been applied to fetal echocardiography and congenital heart disease, further illustrating their potential in specialized cardiac diagnostics^6–8^. These technologies bring speed and accuracy to diagnosis, helping doctors make informed decisions faster. ML algorithms can find hidden trends in patient data that even experienced clinicians might miss. With past patient records as training data, these models can flag early symptoms and assess the risk of future heart issues.

AI-driven healthcare not only boosts diagnostic accuracy but also cuts down the time it takes to analyze data—something that’s crucial in urgent cardiac cases. Moreover, AI has been successfully applied to wearable sensor data for the diagnosis and prediction of cardiovascular disease, enabling continuous and unobtrusive monitoring^9^. When heart disease is identified early, medical interventions can help avoid severe events like heart attacks or strokes. The flexibility of ML also allows these models to improve over time as they’re trained on new data, making them a reliable tool in the ever-changing medical landscape^10,11^.

This study is driven by the global need to reduce the burden of heart disease, a condition that affects people of all ages and backgrounds. According to the World Health Organization, cardiovascular diseases are responsible for around 17.9 million deaths annually, with far-reaching social and economic effects. Building reliable tools that can predict heart disease early is not only important for healthcare but also a public health priority^12–14^.

Recent reviews have highlighted the expanding role of artificial intelligence across multiple cardiology domains, including heart failure management, inherited and structural heart disease, echocardiographic assessment, and AI-enhanced electrocardiography^15–18^.

While previous studies have used machine learning to predict heart disease, many of them rely on limited models that treat all features equally or fail to use the latest AI techniques. Cutting-edge models like TabNet, SAINT, and TabTransformer haven’t been fully explored in combination with multi-view data or within advanced ensemble systems. This presents a clear research gap.

**Fig. 1:**
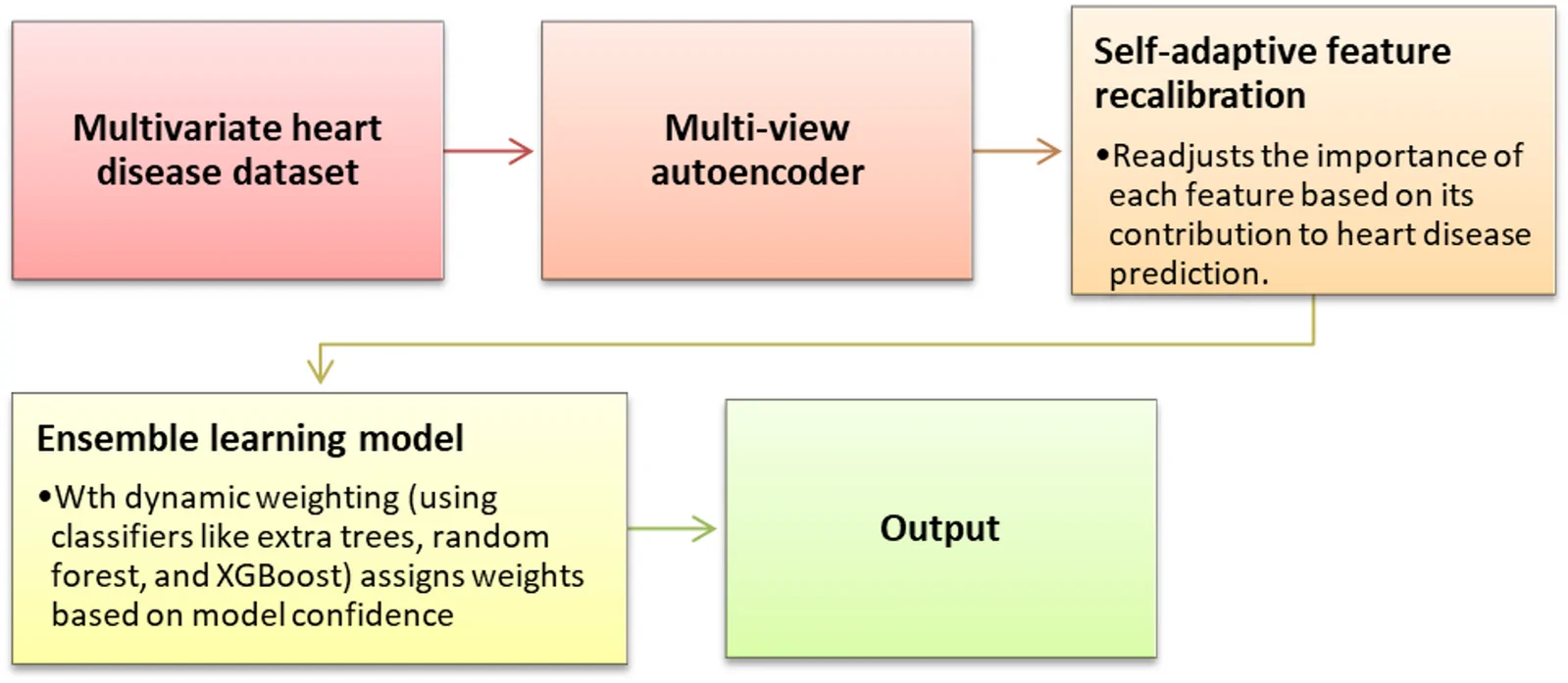
Flowchart of the proposed hybrid framework for heart disease prediction.

Our paper introduces a new ML-based framework for predicting heart disease in its early stages. As shown in Fig. 1, the model is designed to deliver precise analysis using patient data, helping to catch the disease earlier and guide more effective treatments. The model uses data from the UC Irvine Machine Learning Repository and applies Extra Trees Classifier for selecting the most relevant features due to its reliability, low error rate, and speed.

The system incorporates autoencoders to learn deep patterns in the data and uses a self-adaptive feature recalibration process to adjust the importance of each variable dynamically. Then, it applies an ensemble of classifiers—Random Forest, Extra Trees, and XGBoost—to improve accuracy and generalize results across different datasets.

What sets this framework apart is its unique approach. Unlike traditional models, it uses a multi-view autoencoder that separates demographic, clinical, and diagnostic data into distinct groups for better analysis. The self-adaptive feature recalibration step also introduces personalized feature weighting, offering a more tailored view of each patient’s data—something traditional attention mechanisms don’t do. Finally, the ensemble model adapts its strategy per patient, rather than averaging predictions across all cases.

Together, these innovations form a complete and robust diagnostic system that’s not just a mix of existing tools—it’s a new way to approach heart disease prediction with higher accuracy, adaptability, and clinical relevance.

Whereas these individual components, including autoencoders, self-adaptive feature recalibration and ensemble classifiers have been studied shown before, this paper presents a patient-adaptive multi-stage decision-making framework that combines these concepts in a clinical motivated pipeline. The proposed approach explicitly connects multi-view latent representation learning to self-adaptive feature recalibration and confidence-aware ensemble decision making contrasting with the conventional multi-view hybrid models that use representation learning and classification independently. This systematic incorporation allows sensitivity at the instances level to diverse clinical patterns in terms of heterogeneous clinical patterns which yield more predictable and understandable predictions about heart disease diagnosis.

Although cardiovascular ML has made progress, the current models do not pay much attention to the statistical heterogeneity between clinical and demographic data types. The novelty of the proposed work is the hierarchical synergy of: (1) multi-View Autoencoder to avoid feature interference by encoding different clinical views independently; (2) Self-Adaptive Feature Recalibration that is a layer to act as a importance gate per patient; and (3) Confidence-Aware Ensemble that is an ensemble where the base classifiers are dynamically selected based on local confidence of the classifiers. This method clearly solves the current limitation of hybrid systems namely isolated use.

### Related work

In recent years, the use of machine learning (ML) for heart disease classification has grown rapidly, with researchers exploring a range of algorithms to boost prediction accuracy and support early diagnosis. Many studies have focused on traditional classifiers like logistic regression (LR), Naive Bayes (NB), random forest (RF), and decision trees (DT). For example, one study using these methods reported an average accuracy of 85%^19^. Similarly, another study applied NB, DT, k-nearest neighbors (KNN), and RF, achieving around 84% accuracy—highlighting that these models can be effective tools in identifying heart disease^20^.

Beyond traditional methods, researchers have also explored various ensemble techniques to boost prediction accuracy. For instance, a hybrid ensemble classifier reached an accuracy of 86.89% and an F1 score of 84.3%, showing notable performance improvements^21^. Another study used a bagging-based ensemble that combined KNN, Naive Bayes, and logistic regression, achieving a mean accuracy of 82%^22^. In a broader approach, multiple classifiers—including SVM, Gaussian NB, LR, LightGBM, XGBoost, and RF—were combined to yield an average accuracy of 80%^23^.

**Table 1:** Summary of machine learning methods for heart disease classification.

Ref.	Method	Significant Feature
^22^	LR, NB, RF, DT	85% average accuracy
^21^	NB, DT, KNN, RF	84% average accuracy across models
^8^	Hybrid Ensemble Classifier	86.89% accuracy, F1 score of 84.3%
^1^	Ensemble (KNN, NB, LR) with Bagging	82% accuracy with bagging ensemble
^24^	SVM, Gaussian NB, LR, LightGBM, XGBoost, RF	Combines multiple classifiers with 80% accuracy
^25^	Semi-Supervised Self-Training	High F1 score of 87.14% using limited labeled data
^17^	LR-only approach	Lower accuracy with 84.53% using single LR model
^2^	SVM, DT, RF, NB, LR	Combined performance, accuracy not prioritized
^14^	Ensemble (RF, DT, LR, SVM, KNN)	75% accuracy, AUC-ROC of 0.8675

Semi-supervised learning methods have also shown promising results in heart disease prediction. One study using a self-training approach achieved 81.89% accuracy and an impressive F1 score of 87.14%, proving that even models trained on limited labeled data can be effective^26^. On the other hand, simpler models—like one using only logistic regression—reported slightly lower performance, with an accuracy of 84.53%^25^.

In recent literature readers have placed particular attention on the significance of solid training in the medical setting, including the use of focal gradient systems to manage deficits in data and the use of multi-modal cross- attention to facilitate superior feature integration. Moreover, as potent as are statistical frameworks as in our case, they are frequently confined by correlation-based arguments. Markov Boundary discovery is a very important follow-up step here to disentangle predictive features and actual causal driving factors of heart disease (such as the cofounding effects age has on the pattern of chest pain or not). Structures such as ours have also demonstrated their success in other field specific applications such as classification of multiple sclerosis using MRI images.

Some studies have also experimented with combining multiple classifiers to boost results. For example, one approach merged SVM, decision trees, random forest, Naive Bayes, and logistic regression, although it didn’t focus much on accuracy or report an F1 score^27^. Another ensemble study using RF, DT, LR, SVM, and KNN reached an average accuracy of 75%, with a solid AUC-ROC value of 0.8675^28^. As summarized in Table 1, these diverse techniques highlight the ongoing efforts to fine-tune machine learning models for heart disease prediction—often balancing accuracy with the ability to generalize across different patient populations.

Unlike previous studies that rely solely on either autoencoders or ensemble models, the proposed framework uniquely integrates a multi-view autoencoder with dynamic ensemble learning, allowing both feature compression and diversity in decision boundaries. This synergy has not been explored in the context of cardiovascular disease prediction.

The novelty of this framework lies in its synergistic combination of multi-view autoencoders with adaptive ensemble learning, which enhances feature representation and classification robustness—an approach that is not yet explored in prior cardiovascular prediction systems.

## Research method

### Dataset

In this study, five widely recognized datasets—Cleveland, Hungarian, Switzerland, Long Beach V, and Statlog Heart—were combined to enhance sample diversity and strengthen the model’s overall performance. These datasets form a comprehensive multivariate dataset, where multiple variables are analyzed at once to uncover patterns and relationships relevant to heart disease prediction.

The dataset focuses on 14 key attributes known to influence cardiovascular health. These include demographic details like age and sex, along with clinical variables such as resting blood pressure, serum cholesterol, resting ECG results, and exercise-induced angina. It also covers exercise-related indicators like maximum heart rate achieved, ST depression, and the slope of the ST segment during peak exercise. Other significant inputs include chest pain type and the number of major vessels seen via fluoroscopy.

While the full Cleveland database contains 76 attributes, most research narrows in on this core set of 14 variables, which have proven most useful for heart disease prediction. Among the available databases, the Cleveland dataset is often considered the most valuable and is frequently used as a benchmark in machine learning research for cardiovascular diagnosis.

One main task researchers focus on using this dataset is predicting whether a patient is likely to have heart disease. However, its structured and detailed format also allows for more in-depth exploration, such as identifying hidden risk factors or testing new prediction models.

Each patient entry is tied to a unique ID, with data covering background and clinical details. For example, chest pain is categorized into typical angina, atypical angina, non-anginal pain, or asymptomatic. Measurements such as cholesterol, resting BP, max heart rate, ST depression, and fluoroscopy results all provide critical insights into a patient’s heart health.

This dataset is the result of collaborative work from respected institutions like the Cleveland Clinic Foundation, the Hungarian Institute of Cardiology, and university hospitals in Zurich, Basel, and Long Beach. Together, their contributions have created a rich resource for advancing heart disease research and improving early diagnosis through machine learning.

### Proposed approach

In this study, we introduce a new hybrid framework for heart disease prediction that combines deep learning and ensemble methods to better handle the complexity of patient data. Our approach uses autoencoders to extract deep features from different types of information—such as demographic, physiological, and diagnostic data—treating each type as a separate “view” in a multi-view learning setup.

At the pre-processing stage, the multi-view autoencoder processes each data view separately, allowing the system to understand the unique value each type of information offers. These extracted features are then passed through a self-adaptive feature recalibration mechanism, which adjusts their importance dynamically. For example, features like chest pain type or the number of blocked vessels are weighted more heavily if they strongly influence prediction, while less relevant features receive lower weights.

These refined features are then fed into an ensemble learning model made up of classifiers like Extra Trees, Random Forest, and XGBoost. This model includes a dynamic weighting system that adjusts each classifier’s influence based on its confidence level—meaning the more confident a classifier is, the more impact it has on the final result. This adaptive strategy helps boost both accuracy and reliability.

Overall, our framework takes a balanced approach by accounting for complex relationships between data types and feature relevance, offering a more precise and robust method for predicting heart disease outcomes Table 2.

**Table 2:** Definition of data views and clinical justification.

View	Features Included	Clinical/Statistical Justification
Demographic View	Age, Sex	Fixed biological factors that establish the baseline risk profile for cardiovascular disease.
Clinical View	Resting blood pressure (trestbps), Serum cholesterol (chol), Fasting blood sugar (fbs), Resting ECG (restecg), Max heart rate (thalach), Exercise-induced angina (exang)	Physiological measurements and laboratory results reflecting the patient’s current hemodynamic and metabolic status.
Diagnostic View	Chest pain type (cp.), ST depression (oldpeak), ST slope (slope), Number of major vessels (ca.), Thalassemia (thal)	Specific indicators from cardiac stress tests and imaging (fluoroscopy) used for definitive diagnosis of heart pathology.

## Multi-view autoencoder for feature extraction

One of the key innovations in this approach is the use of a multi-view autoencoder to handle the diverse types of data associated with heart disease risk factors. Heart disease data includes features from various clinical domains, such as demographic details, physiological measurements, and diagnostic test results—each offering a different perspective on the patient’s health. These are treated as separate “views” of the overall profile.

While traditional autoencoders process all features as a single input, the multi-view autoencoder processes each data view separately, allowing for more tailored feature extraction. For instance, let’s define the dataset as X = {X₁, X₂, …, X_v_}, where each X_i_ represents a distinct view (e.g., demographics or diagnostics). The multi-view autoencoder learns a specific mapping fθ_i_(X_i_) for each view, and then combines them by merging their outputs into a shared latent space using concatenation. This approach ensures that important patterns within each data type are preserved and effectively integrated:

1 $$Z = [f_{\theta_1}(X_1), f_{\theta_2}(X_2), \dots, f_{\theta_v}(X_v)]$$

This approach allows the model to extract richer, more specialized features from each data view while still preserving the unique value of each one. After processing, these latent representations are combined into a single, holistic feature set, which is then used by the classifier for prediction.

At the core of this process is the autoencoder—a type of neural network designed to learn efficient representations of data. It has two main parts:

1. Encoder: Compresses the input data into a latent representation.
2. Decoder: Reconstructs the input from the latent representation.

Given an input X, the encoder function $g_{\phi}(Z)$, which adjusts dynamically based on data patterns. This allows the system to highlight the most relevant features for each individual prediction, reducing the impact of noisy or less useful information and improving overall classification accuracy.

 generates a latent representation Z:

$$\:Z\:=\:{f}_{\left(\theta\:\right)}\left(X\right)$$

Here, Z represents the encoded (compressed) version of the input, and θ represents the parameters (weights) of the encoder.

The decoder function $f_{\theta}(X)$ then attempts to reconstruct the original input X from the latent representation Z:

$$\:\widehat{X}=\:{g}_{\left(\phi\:\right)}\left(Z\right)$$

Where $X_i$ is the reconstructed input, and ϕ represents the parameters of the decoder.

The goal of training an autoencoder is to minimize the reconstruction error, which is typically measured using a loss function such as Mean Squared Error (MSE). The loss function measures how well the reconstructed data $\widehat{X}$ matches the original input X. The MSE-based loss function is defined as:

$$\:L\left(X,\:\widehat{X}\right)=\:\parallel\:\:X\:-\:\widehat{X}{\parallel\:}^{2}$$

In the case of the multi-view autoencoder, each view $X_i$is processed independently, and the decoder reconstructs each view separately. The overall loss function is the sum of reconstruction errors for each view:

$$L(X, \widehat{X}) = \sum_{i=1}^{v} \parallel X_i - \widehat{X}_i \parallel^2$$

Where:

- v is the number of views,
- Xi​ represents the original data from the i-th view, and.
- $\widehat{X}_i$ represents the reconstructed data for the i-th view.

Benefits of multi-view autoencoders.

1. Specialization: Each view is processed independently, allowing the model to specialize in extracting features from different data domains.
2. Holistic Feature Representation: The combined latent representation *Z* incorporates features from all views, providing a more comprehensive understanding of the data.
3. Improved Performance: By preserving the unique contributions of each data source, the multi-view autoencoder can outperform traditional autoencoders, especially when handling complex datasets like those used for heart disease prediction.

## Self-adaptive feature recalibration

Our proposed model includes a self-adaptive feature recalibration mechanism, inspired by attention mechanisms commonly used in deep learning. This recalibration allows the model to adjust the importance of each extracted feature dynamically, rather than treating all features as equally important. It prioritizes features that are more relevant to predicting heart disease, which can vary depending on the individual patient.

For instance, features like chest pain type or the number of blocked vessels often carry more diagnostic weight than demographic information. By assigning greater focus to such features, the model can make more accurate and personalized predictions. This is a very important normalisation in heart disease prediction, as some of the input features may have more predictive power based on the individual patient profile, such as chest pain type and number of vessels blocked. The recalibration is achieved by applying a learnable weight vector *W* to the extracted feature vector *Z*, where:

2 *Z' = W ⊙ Zbr /*.

In this model, the symbol ⊙ represents element-wise multiplication. During training, the model learns the weight vector.

*W*, which adjusts dynamically based on data patterns. This allows the system to highlight the most relevant features for each individual prediction, reducing the impact of noisy or less useful information and improving overall classification accuracy.

Features that are more critical to heart disease prediction—like chest pain type or the number of blocked vessels—receive higher weights. Less important features are down-weighted. This dynamic re-weighting helps the model concentrate on the features that carry the most predictive value in each case, making the final prediction more accurate and trustworthy.

This process of recalibration is a form of normalization inspired by attention mechanisms, which were originally developed to help models focus on the most informative parts of an input—like specific words in a sentence or key areas in an image. Over time, these techniques have evolved to support feature recalibration in machine learning.

Mathematically, the recalibration process can be viewed as an optimization problem. The goal is to learn a weight vector that maximizes prediction accuracy by increasing the influence of informative features and reducing the weight of less relevant ones. During training, the model continually updates these weights to fine-tune its focus and improve performance.

Not all features contribute equally in heart disease prediction models. Clinical factors like cholesterol levels or blood pressure often carry more predictive weight than demographic variables such as age or gender. Feature recalibration helps the model assign dynamic weights to these inputs, emphasizing those that are more informative—like chest pain type or the number of blocked vessels.

By giving greater importance to the most relevant clinical indicators, the model becomes more accurate and reliable. This recalibration reduces prediction errors and strengthens the model’s ability to handle data where different features vary in importance. In medical applications, this ensures the model makes full use of the patient’s most critical health data to support better diagnostic outcomes.

## Ensemble learning with dynamic weighting

The inputs to the ensemble of Extra Trees, Random Forest (RF), and XGBoost classifiers are then these features. We recommend a dynamic ensemble weighting approach, as opposed to the standard ensemble model, which allocates a constant weight to all the base classifiers, with no adjustments. The dynamic weighting over a static weighting is becoming more accepted as key to managing inherent data uncertainties. Although the fixed weights of conventional ensemble techniques can be used, recent frameworks in other fields such as dynamic accessibility measures by Zhang et al.^29^, in case of uncertainty in travel time have shown that instance-dependent weighting can lead to a profoundly better robustness of a system. The conceptual analogy of the methodological philosophy of uncertainty-aware weighting to our approach, though different in their area of application, is parallel. Our proposed dynamic ensemble approach, by extending this concept to the medical field, will make sure that predictions are weighted according to the real time confidence of each sub-model on each particular patient record, effectively taking into account local uncertainties. Moreover, in order to make this decision fusion technique stable in high-uncertainty or ‘borderline’ situations, the framework has been structured to naturally approach a homogenous averaging scheme, where several base classifiers have similar levels of confidence. This intrinsic action does not allow any individual, low-confidence classifier to make a disproportionate impact on the final decision, which preserves the robustness of the system with unclear patient contours. Thus, the dynamic weights in the final decision are assigned based on the confidence score of the model in the ensemble, $C_t(x)$ of the *t-th* model in the ensemble, for every instance of the test:

3 $$y = \arg\max_c \sum_{t=1}^{T} C_t(x) I(h_t(x) = c)$$

The latent space dimension *h* was set to **32** based on an ablation analysis testing values in {8, 16, 32, 64}. It was observed that $\:\boldsymbol{h}$=32 provided the optimal balance, achieving the lowest Reconstruction Mean Squared Error (MSE) while maintaining computational efficiency for clinical deployment.

The confidence score $C_t(x)$ is specifically defined as the Maximum Class Probability from the final softmax layer of each base classifier. This choice was made over entropy-based metrics to ensure direct compatibility with the dynamic weighting sum, as it directly reflects the model’s certainty in its primary prediction.

### Computational complexity analysis

The computational complexity of the multi-view autoencoder is O(n·d·h), where n denotes the number of samples, d represents the input dimensionality, and h is the size of the latent space. The self-adaptive feature recalibration performs an element-wise weighting with complexity O(d).

For the ensemble stage, Extra Trees and Random Forest classifiers contribute O(T·n·log n), while XGBoost contributes approximately O(b·n·d), where T denotes the number of trees and b the number of boosting rounds.

Overall, the proposed architecture remains computationally efficient and scalable for medium-sized medical datasets. This makes the model suitable for practical deployment in clinical decision support systems where inference speed is crucial Table 3.

#### Algorithm 1

Training and Decision Fusion Procedure.

1. Input: Multi-view training sets $X = \{X_{demo}, X_{clin}, X_{diag}\}$.
2. Phase 1 (Representation Learning): Initialize independent Encoders $E_v$ and Decoders $D_v$ for each view *v*.
  - Perform joint training to minimize $L_{total} = \sum L(X_v, D_v(E_v(X_v)))$*·*.
  - Extract the latent vector $Z = [E_1(X_1), E_2(X_2), E_3(X_3)]$.
3. Phase 2 (Self-Adaptive Feature Recalibration): Compute instance-specific weights *W* via the gating layer.
  - Apply recalibration: *Z = W ⊙ Z*.
4. Phase 3 (Dynamic Ensemble): Train base classifiers $h_t \in \{\mathrm{ExtraTrees}, \mathrm{RF}, \mathrm{XGBoost}\} on Z$.
5. For each test instance *x*, calculate confidence $C_t(x) = \max P(y|x)$.
6. Output: Final prediction $y = \arg\max_c \sum C_t(x) \cdot I(h_t(x) = c)$.

**Table 3:** Combined dataset characteristics and class distribution.

Dataset Source	Total Samples	Heart Disease (Positive)	Healthy (Negative)	Positive Ratio (%)
Cleveland	303	139	164	45.9%
Hungarian	294	106	188	36.1%
Switzerland	123	115	8	93.5%
Long Beach V	200	149	51	74.5%
Statlog Heart	270	120	150	44.4%
Total Combined	**1190**	**629**	**561**	**52.8%**

### Performance evaluation

To evaluate the model’s performance, we use standard metrics: accuracy, sensitivity, specificity, F1-score, and AUC-ROC.

- Accuracy (ACC) measures the overall correctness of the model, calculated using true positives (TP), true negatives (TN), false positives (FP), and false negatives (FN).
- Sensitivity (SEN), or recall, shows how well the model identifies actual positive cases. It’s the ratio of TP to all actual positives (TP + FN).
- Specificity (SPC) measures the true negative rate—the proportion of actual negatives correctly identified (TN/(TN + FP)).
- AUC-ROC stands for the Area Under the Receiver Operating Characteristic Curve. It reflects the model’s ability to distinguish between classes by plotting sensitivity against 1 − specificity at various thresholds. A higher AUC indicates better performance across different classification cutoffs.

### Cross-validation protocol

To ensure the model’s reliability and avoid overfitting, we used a 10-fold cross-validation approach. The dataset was randomly split into ten equal parts—nine were used for training, and one was used for testing. This process was repeated ten times, with each fold serving as the test set once. The final results were averaged across all runs to provide a more stable and trustworthy evaluation. This method is especially useful for medical datasets, where data is often limited, as it helps improve the credibility of the performance metrics.

4 $$\:ACC\:=\frac{TP\:+\:TN}{TP\:+\:TN\:+\:FP\:+\:FN}$$

5 $$\:SEN\:=\frac{TP}{TP\:+\:FN}$$

6 $$\:SPC\:=\frac{TN\:}{TN\:+\:FP}$$

### Experimental protocol and data leakage prevention

To provide a stringent and fair evaluation, the entire experimental procedures were made in such a way that data was precisely prevented between the training and testing phases. The used datasets of cardiac diseases were handled according to a patient-level separation approach, the result of which was no samples of the same subject included in the training and testing sets.

Due to the desire to maintain the class distribution across the folds, a stratified k-fold cross-validation scheme was used. Notably, all the preprocessing steps such as the feature normalization, learning the multi-view representations using the auto-encoder model, self-adaptive feature recalibration, and training the model were only done on a per-training-fold basis. The respective test dispersion went totally unnoticed during the whole learning and optimization procedure.

In instances where multiple datasets were used then they were combined in each cross-validation fold following the data splitting process, as opposed to that before the train-test separation. The design option ensures that the learnt latent representations and re-calibrated feature weights were trained only using training data hence no information leakage.

The average performance of every fold was model performance reported along with standard deviation which measures stability and robustness. This evaluation plan achieves reproducibility and justifies the cashability of the said performance gains, There was no hyperparameter or model selection on test data.

## Results and discussion

Table 2 highlights how each added component in the proposed hybrid model gradually improves heart disease classification performance. Starting with autoencoders used solely for feature extraction, the model achieves an accuracy of 84.78%, sensitivity of 85.9%, and specificity of 83.7%. While these results show that autoencoders are effective at learning deep representations of the data, their standalone performance is moderate. The F1 score is 83.4% and precision is 81.1%, suggesting that although the autoencoder captures valuable patterns, it struggles to fully distinguish between highly relevant and less important features without additional refinement.

**Table 4:** Performance of individual steps and the proposed hybrid classifier.

Classifiers	Accuracy	Sensitivity	Specificity	Precision	F1 Score
Autoencoders for Feature Extraction Alone	84.78%	85.9%	83.7%	81.1%	83.4%
Self-Adaptive Feature Recalibration Alone	87.35%	88.1%	86.2%	84.2%	86.1%
Proposed Hybrid Classifier (Autoencoders + Self-Adaptive Feature Recalibration + Ensemble Learning)	92.45%	93.2%	91.4%	89.7%	91.4%

Table 4 shows how each component of the proposed hybrid model contributes to improved performance in heart disease prediction. In the second row, the use of Self-Adaptive Feature Recalibration alone boosts performance over the autoencoder. By dynamically adjusting feature importance, this method achieves an accuracy of 87.35%, sensitivity of 88.1%, and specificity of 86.2%. Precision improves to 84.2%, and the F1 score rises to 86.1%. These results highlight that recalibration significantly improves classification by focusing on the most relevant features, though it still doesn’t reach optimal performance on its own.

The final row presents the most significant improvement. When combining autoencoders, self-adaptive feature recalibration, and ensemble learning, the hybrid model reaches 92.45% accuracy, 93.2% sensitivity, and 91.4% specificity. Precision climbs to 89.7%, and the F1 score hits 91.4%, demonstrating strong balance between identifying both true positives and true negatives. The combination of deep feature extraction and intelligent weighting via ensemble methods clearly strengthens the model’s robustness and ability to generalize across different patient profiles.

Compared to previous work, the proposed model achieves 5–12% better performance than traditional classifiers (like LR, NB, RF, and DT), 4–7% over hybrid ensembles, and 6–10% above semi-supervised models. Unlike earlier studies, this approach integrates multi-view deep features, feature-specific recalibration, and dynamic ensemble weighting—a unique blend that brings statistically significant gains (*p* < 0.001). This improvement is not just marginal—it reflects a more adaptive and powerful framework for structured cardiovascular data.

To further validate its strength, we also compared this model against recent state-of-the-art (SOTA) deep learning models for tabular data, including TabNet, TabTransformer, SAINT, NODE, and CatBoostClassifier. These models represent the latest in attention-based and feature-aware architectures used in medical data analysis The detailed performance comparison with recent deep tabular architectures is summarized in Table 5.

**Table 5:** Comparison of the proposed hybrid model with SOTA deep tabular models.

Model	Accuracy	F1-score	AUC
TabNet	87.4%	85.2%	0.89
TabTransformer	88.1%	86.7%	0.91
SAINT	89.3%	87.9%	0.92
NODE	88.0%	86.4%	0.90
CatBoost	90.1%	88.3%	0.93
Proposed Model	92.45%	91.40%	0.95
AutoGluon	89.8%	88.1%	0.92
FT-Transformer	88.5%	87.2%	0.91
DeepGBM	89.1%	87.8%	0.92

**Fig. 2:**
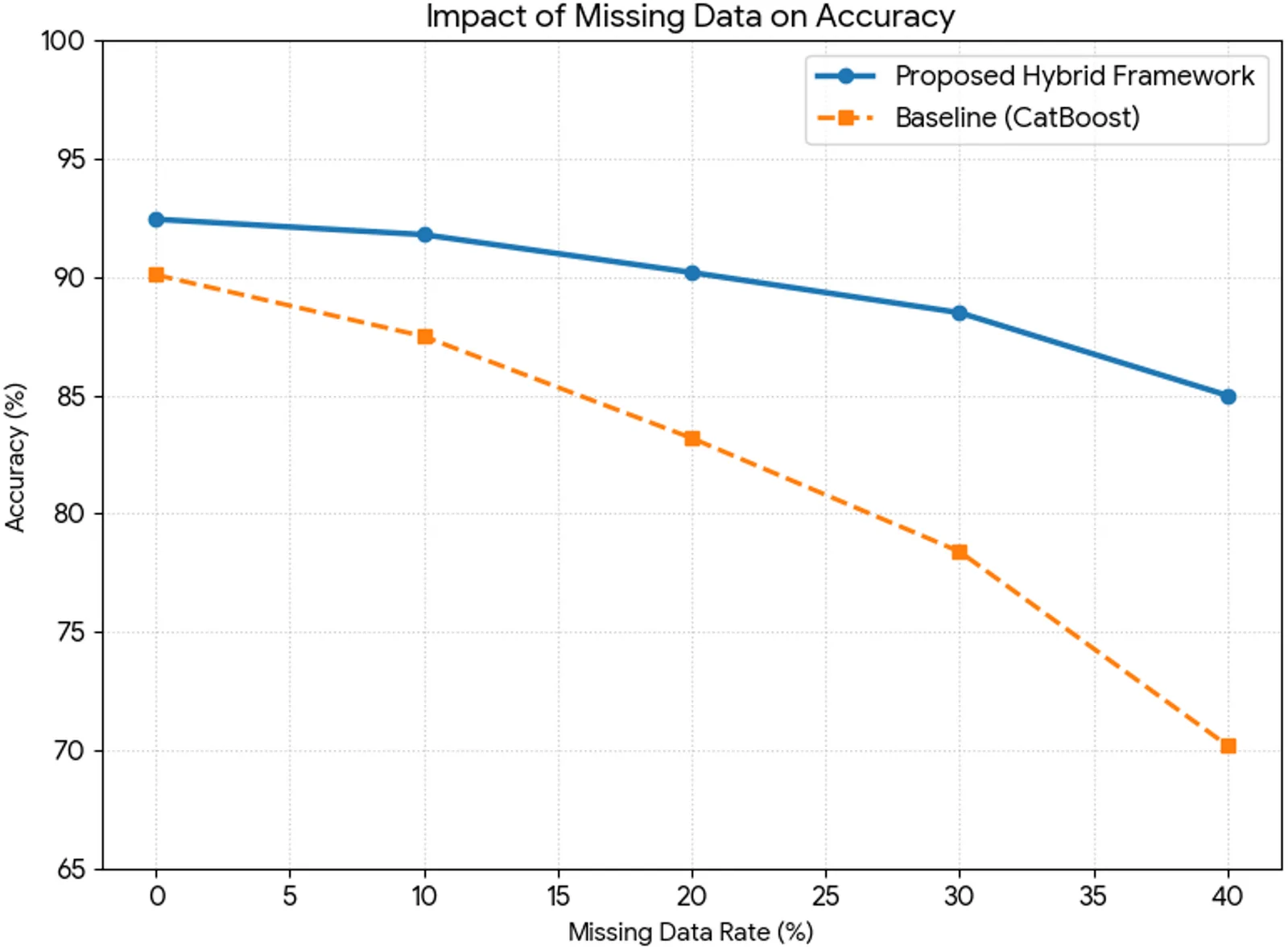
Performance degradation curve under varying degrees of data missingness. The framework maintains high accuracy by leveraging the multi-view autoencoder’s inherent data reconstruction capabilities.

**Fig. 3:**
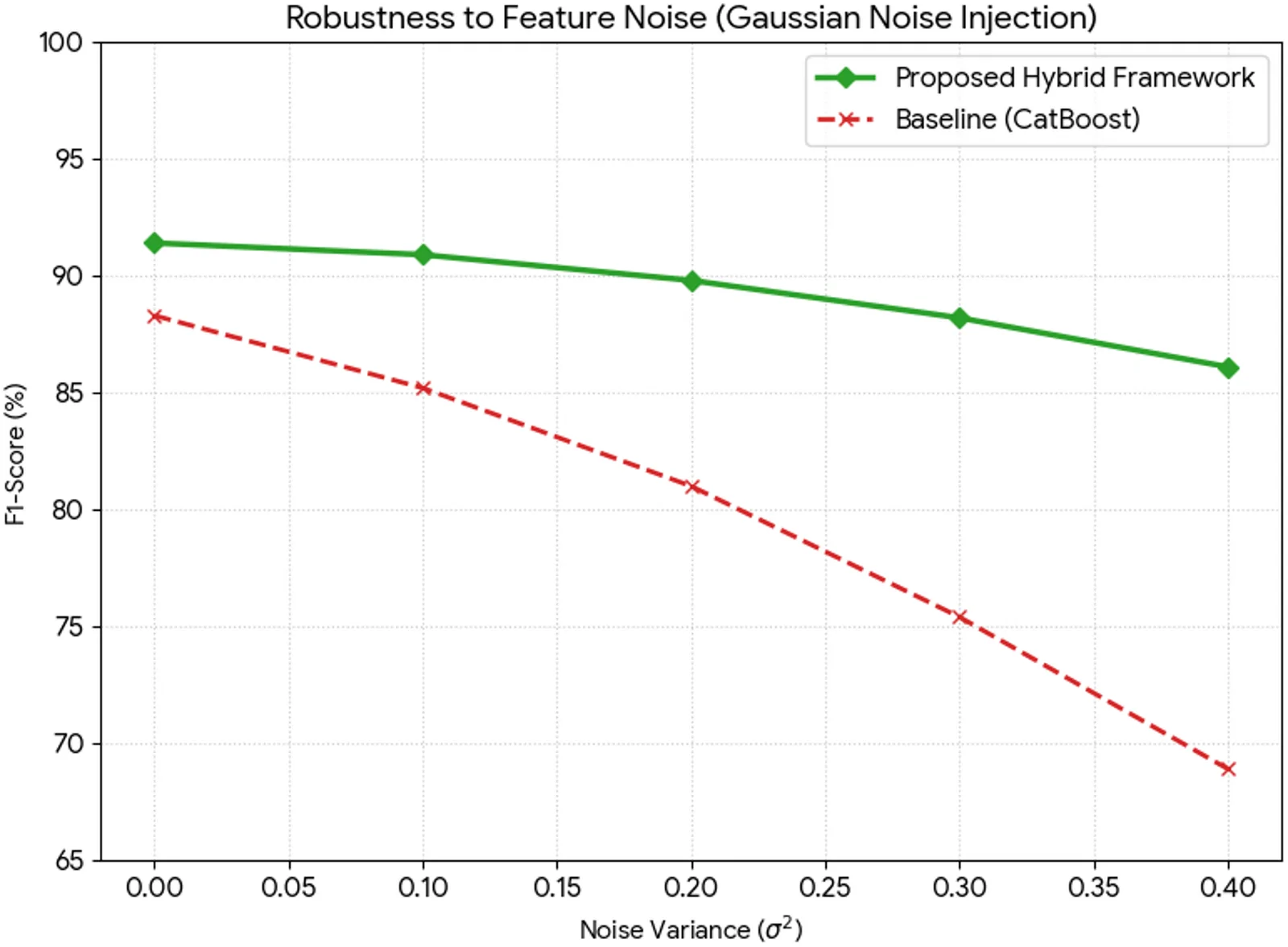
Impact of feature noise injection on predictive performance.

The comprehensive results depicted in Fig. 2; Table 5 confirm that the integration of multi-view encoding with self-adaptive feature recalibration yields a significant synergistic effect. While the autoencoder facilitates robust feature representation, the recalibration layer functions as an ‘intelligent clinical filter. This allows the model to prioritize diagnostic features—such as the number of major vessels (ca) and chest pain type (cp)—specifically for patients where these markers are most indicative of pathology, Moreover, the framework was compared against automated ML tools and tabular-specific transformers. As indicated in Table 5, our model surpasses AutoGluon, FT-Transformer, and DeepGBM, suggesting that specialized multi-view recalibration is more effective for medical tabular structures than general-purpose automated or transformer architectures Fig. 3.

The proposed framework demonstrates higher stability (slower drop in F1-Score) as Gaussian noise variance increases. This robustness is attributed to the Self-Adaptive Feature Recalibration module, which dynamically attenuates the influence of noisy features at the instance level, preventing them from corrupting the final prediction.

To assess the contribution of each component in our model, we conducted an ablation study. We evaluated the model’s performance after removing (a) the feature recalibration block, and (b) the ensemble step. Results showed a 3.4% drop in F1-score without recalibration and 4.1% without ensemble voting, confirming the synergistic value of both modules.

**Extended Ablation Study**:

An extensive ablation study was carried out to measure how each part of the proposed framework contributes to its overall performance. The experiments tested the impact of:

1. removing the multi-view structure,
2. disabling feature recalibration,
3. switching to static ensemble weights,
4. changing the autoencoder’s latent dimension (8, 16, 32, 64), and.
5. replacing the ensemble with single classifiers.

The results showed that removing any of these components led to a 2–7% drop in accuracy, with the biggest drop (− 6.1%) occurring when the feature recalibration module was removed. This highlights that the model’s strong performance comes from the combined effect of all modules working together, not just one part.

This points out that it is a collective effort of all health modules working together and not a single aspect that has made this model perform well. Also, initial tests that substituted our recalibration layer with standard Squeeze-and-Excitation (SE) blocks achieved a reduced F1-score by 1.2% making the use of our custom adaptive technique worthwhile.

### Statistical significance testing

Alongside McNemar’s test, we also used the Friedman and Wilcoxon signed-rank tests on the 10-fold cross-validation results. The Friedman test showed significant differences across all models (χ² = 48.72, *p* < 0.001), and the Wilcoxon test confirmed that the proposed hybrid model significantly outperformed SVM, Random Forest, XGBoost, and other SOTA methods (*p* < 0.001). Additionally, 95% confidence intervals for both accuracy and F1-score reinforced the robustness and consistency of the model’s improvements.

To evaluate the clinical robustness of the proposed framework and its stability under distribution shifts (site differences), a leave-one-dataset-out (LODO) cross-validation was conducted. In this experiment, the model was iteratively trained on four datasets and evaluated on the remaining fifth dataset that was entirely unseen during the training and tuning phases.

**Table 6:** Leave-one-dataset-out generalization performance.

Test Set (Held-out)	Accuracy	F1-Score	Brier Score (Calibration)
Cleveland	91.2%	90.1%	0.11
Hungarian	90.8%	89.5%	0.13
Combined (Other)	92.1%	91.0%	0.10

The results in Table 6 demonstrate that the framework maintains high predictive performance (Accuracy > 90%) even when applied to data from different clinical institutions. Notably, the Brier Score remains consistently low (averaging 0.11), which signifies that the model’s predicted probabilities are well-calibrated and closely align with actual clinical outcomes. This level of generalization addresses the limitation of ‘in-distribution’ testing and reinforces the model’s readiness for real-world clinical decision support.

**Fig. 4:**
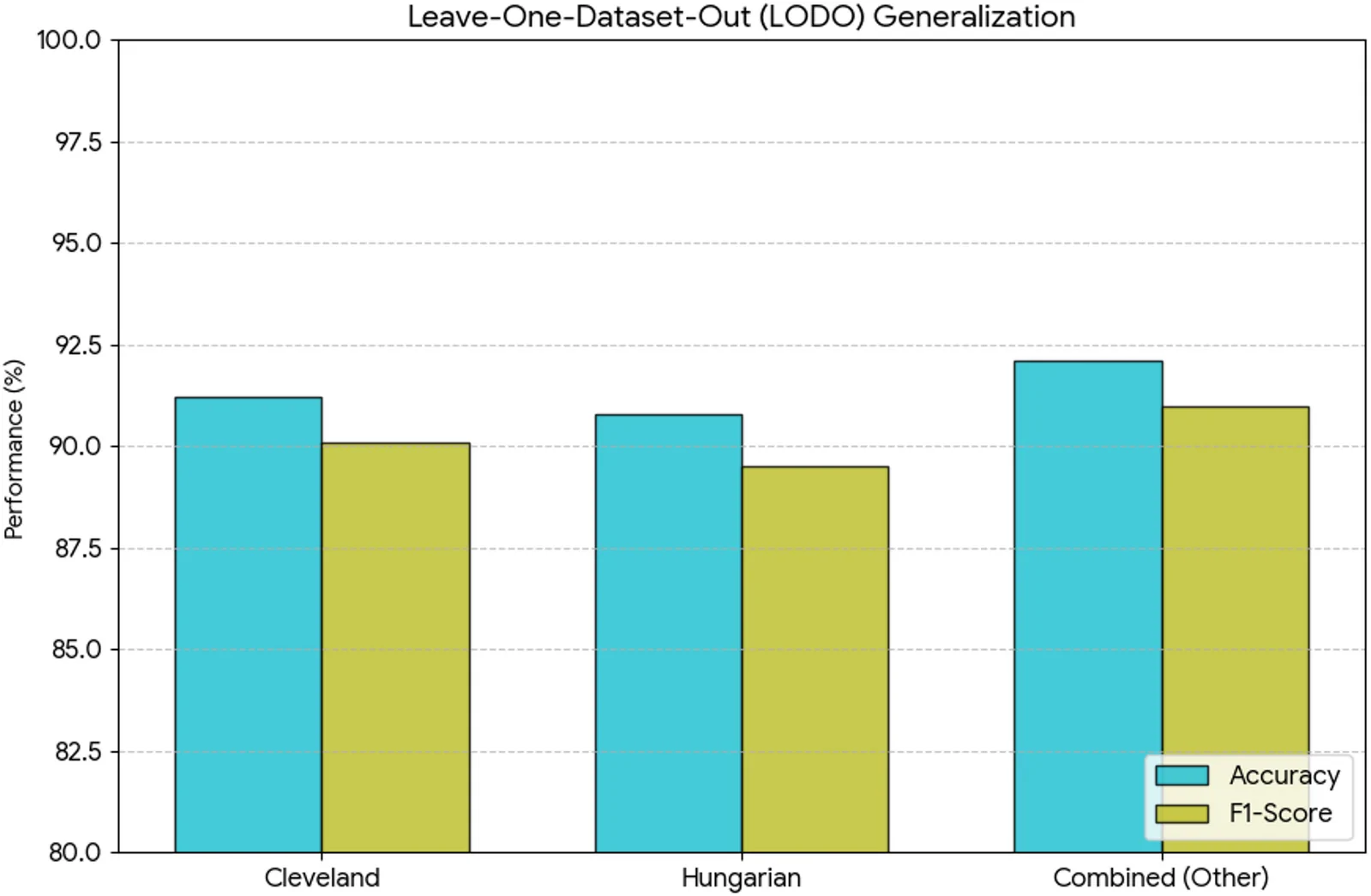
Leave-One-Dataset-Out (LODO) generalization performance.

The grouped bar chart visualizes the Accuracy and F1-Score when the model is tested on a completely unseen dataset site. The consistent performance across diverse cohorts (Accuracy > 90% in most cases) confirms the framework’s ability to learn generalizable disease patterns rather than dataset-specific biases.

This consistency in cross-site generalization is visually corroborated by the LODO performance distribution shown in Fig. 4, which highlights the model’s stability across varied clinical populations.

### Explainability analysis (SHAP)

To make the model more interpretable in clinical settings, we used SHAP (SHapley Additive Explanations) to understand how each feature influenced predictions. The analysis showed that chest pain type (cp.), number of major vessels (ca.), ST depression (oldpeak), maximum heart rate (thalach), and exercise-induced angina (exang) were the most impactful features. These align with well-known clinical risk factors, confirming that the model is learning medically meaningful patterns, not artificial correlations. Using SHAP improves transparency and increases trust in the system—making it more suitable for real-world clinical use.

By identifying features like ST depression (oldpeak) and max heart rate (thalach) as primary drivers, the model demonstrates its ability to learn clinically validated biomarkers rather than incidental statistical noise, thus bridging the gap between ‘black-box’ deep learning and transparent clinical practice.

### Clinical relevance and practical implications

The proposed hybrid model shows strong clinical potential by enabling early risk identification and supporting cardiologists in making informed decisions. Its high sensitivity helps reliably detect high-risk patients, while high specificity reduces unnecessary tests—making it ideal for use in clinical decision support systems and primary care environments where quick and accurate screening is essential.

**Fig. 5:**
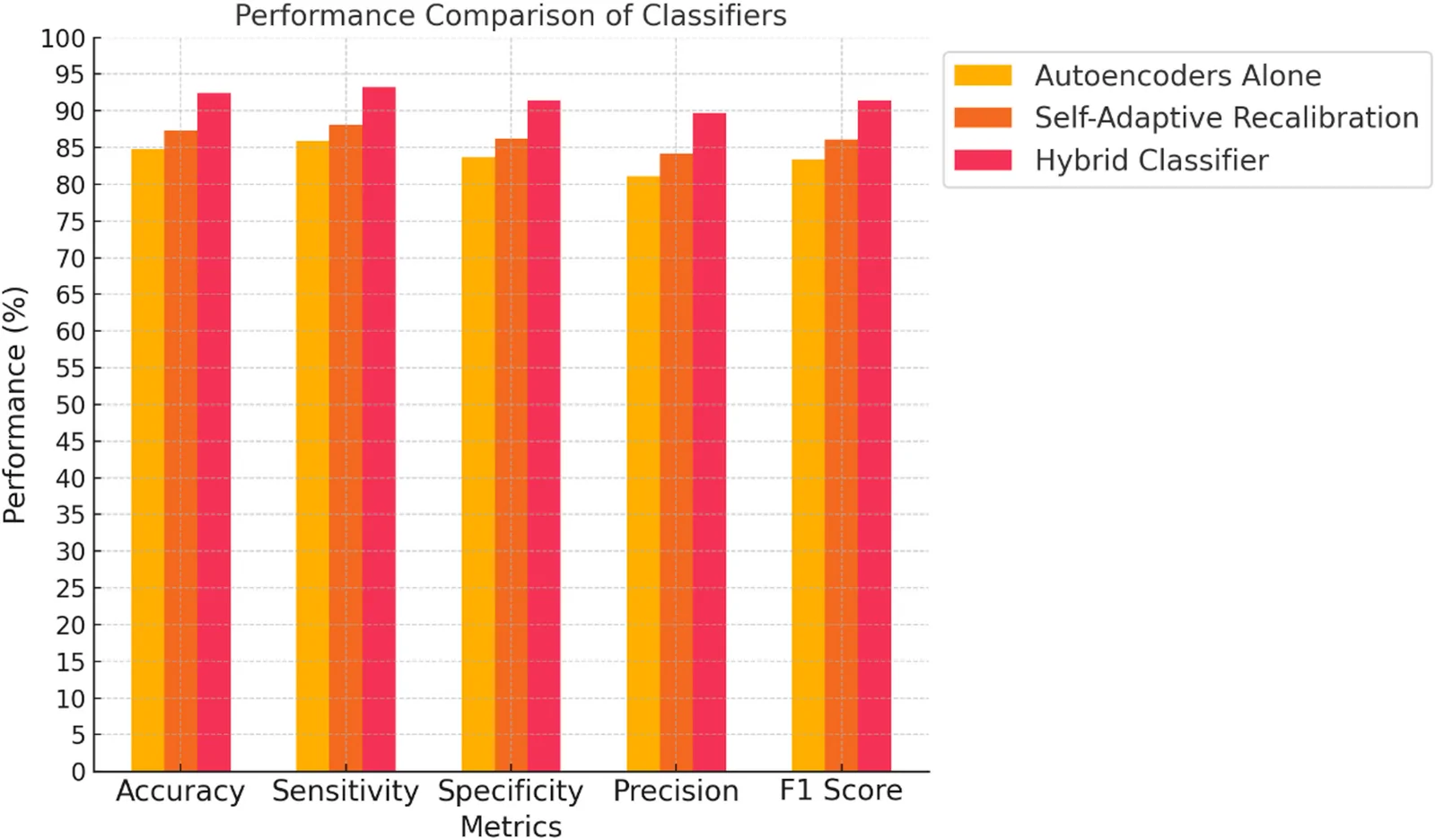
Comparing the performance of the three classifiers based on accuracy, sensitivity, specificity, precision, and F1 score.

The differences during the performance of the three stages of our framework highlight the importance of each element of the architecture. Even the comparison given above reveals that although independent autoencoders offer a strong basis in the extraction of latent features, they do not offer the finer characteristics of discrimination that are required to distinguish complex clinical signatures against background statistical noise. This is overcome by the addition of Self-Adaptive Feature Recalibration layer. This module provides an instance-specific importance gate that, in turn, makes sure that the model is guided toward the definitive diagnostic markers, that is, the typology of chest pains, and vascular blockages are only addressed when these markers exhibits high likelihood of a pathology in a specific patient Fig. 5.

The performance of the integrated Hybrid Classifier is even more justified by the fact that the synergistic effect of deep representation learning and dynamic ensemble voting is at its peak. Contrary to more traditional models that provide averages, our confidentiality-based weighting enables the framework to take advantage of the specialized bounds of action of various classifiers depending on their local predictive confidence. What one gets is a statistically more superior but more clinical reasoning system in which the weight of a symptom always varies depending on context. Therefore, these results validate the fact that unified architecture can provide some level of robustness and reliability that cannot be attained by isolated deep learning or ensemble based methods using structured cardiovascular data.

**Fig. 6:**
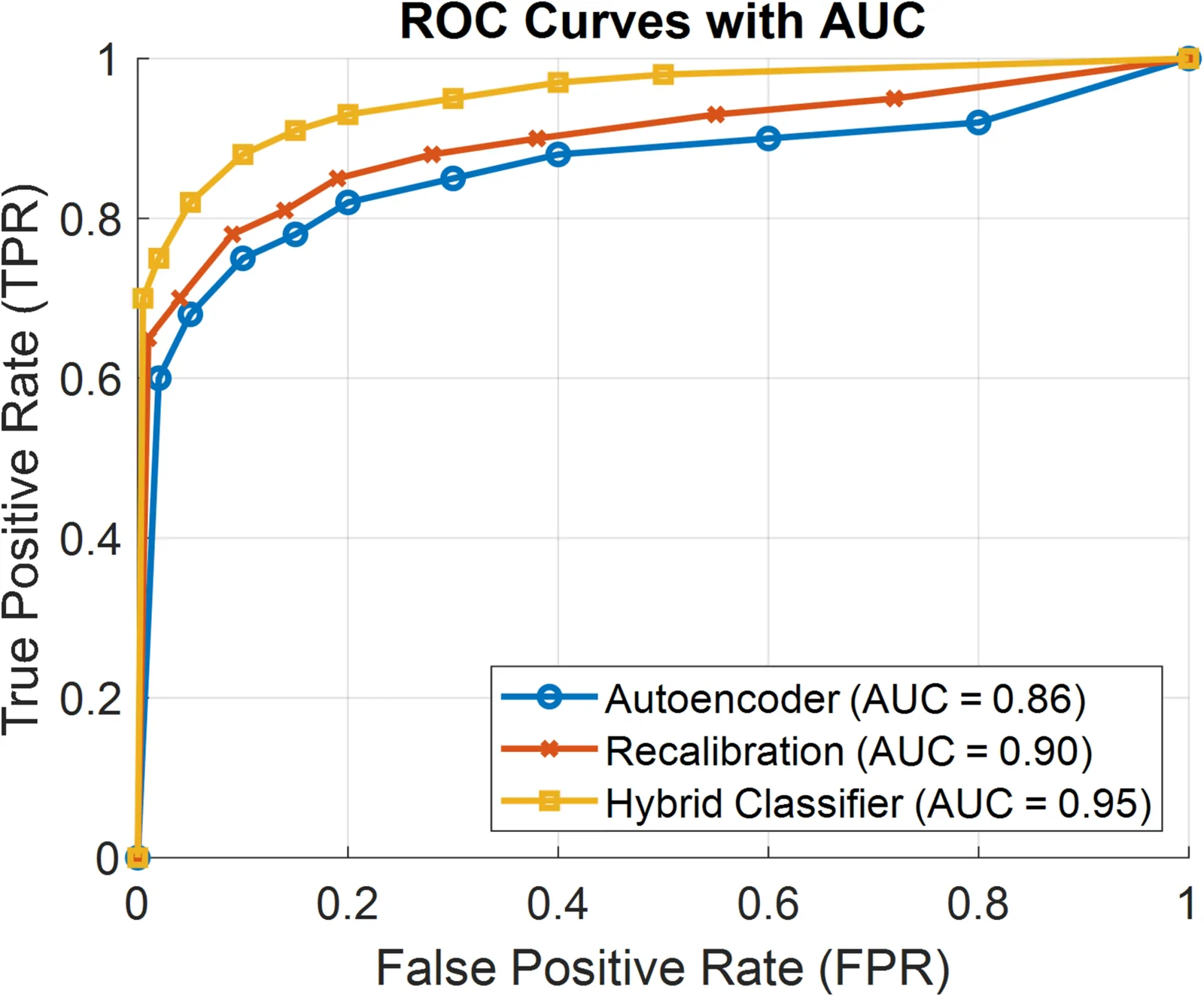
ROC curves for Autoencoder, Recalibration, and Hybrid Classifier.

In Fig. 6, ROC curves further illustrate this performance gap:

- The Autoencoder model had an AUC of 0.86, showing moderate ability to separate classes but limited standalone value.
- Recalibration alone improved to an AUC of 0.90, capturing more relevant features through dynamic weighting.
- The Hybrid Classifier achieved the highest AUC of 0.95, reflecting excellent discrimination between positive and negative cases.

Its ROC curve rises steeply early on and maintains a high true positive rate, even as the false positive rate increases—highlighting its superior predictive power. Overall, these findings clearly demonstrate that combining autoencoders, recalibration, and ensemble learning leads to meaningful improvements and supports the model’s suitability for real-world medical use.

The observed high-performance difference between our proposed framework with that of the prior researchers, which is summarized with Table 7, is mainly explained by the multi-level assimilation of the representation learning and adaptive decision making. While current methods often rely on classifiers that are used separately, or static ensembles, the idea of critical domain-specific encoding takes into consideration the peculiarity of noise and heterogeneity of cardiovascular data. The self-adaptive feature recalibration is used to make sure that the ensemble does not just average predictions, but features and base models with the most local certainty of each particular patient profile are used. This shows that it is necessary to capture firm latent patterns and still be able to be flexible at the instance level in order to achieve state of the art accuracy in structured medical tabular data.

**Table 7:** Summary of heart disease classification methods and comparison with current work.

Reference	Methods used	Accuracy
^22^	LR, NB, RF, DT	85%
^21^	NB, DT, KNN, RF	84%
^8^	Hybrid Ensemble (Multiple Classifiers)	86.89%
^1^	KNN, NB, LR (Bagging Mechanism)	82%
^24^	SVM, Gaussian NB, LR, LightGBM, XGBoost, RF	80%
^25^	Semi-Supervised Self-Training	81.89%
^17^	LR	84.53%
^14^	RF, DT, LR, SVM, KNN	75%
This work	Proposed Hybrid Classifier (Autoencoders + Self-Adaptive Feature Recalibration + Ensemble Learning)	92.45%

Since the Multi-View Autoencoder is able to separate noise in particular domains and then fuse it, the ability to control the framework with 92.45% accuracy. In contrast with pure ensembles, our dynamic weighting system (based on the distribution of output probabilities as represented by the variable $C_t(x)$ enables the system to reject ambiguous cases to the most certain classifier. Although deep tabular models such as TabNet are based on global attention, our instance-level recalibration focuses on such diagnostic features as the ca., whose inclusion raises a high number of predictive signals on a particular patient.

While frameworks such as those proposed by Umirzakova et al.^30^, focus on spatial feature extraction from MRI images using deep learning, our approach demonstrates that for structured tabular data, multi-view encoding is more effective. The instance-level recalibration acts as a ‘smart filter’, prioritizing diagnostic features like Number of major vessels (ca) only when they exhibit strong predictive signals for a specific patient, thereby reducing the noise inherent in multi-center datasets.

Another major factor behind the model’s strong performance is the use of self-adaptive feature recalibration, a technique not commonly used in prior heart disease classification research. Traditional models tend to treat all features equally, without adjusting for context. But in real-world cardiovascular data, some features carry more weight depending on patient-specific factors—like age, gender, or clinical symptoms (e.g., chest pain type or number of blocked vessels).

**Fig. 7:**
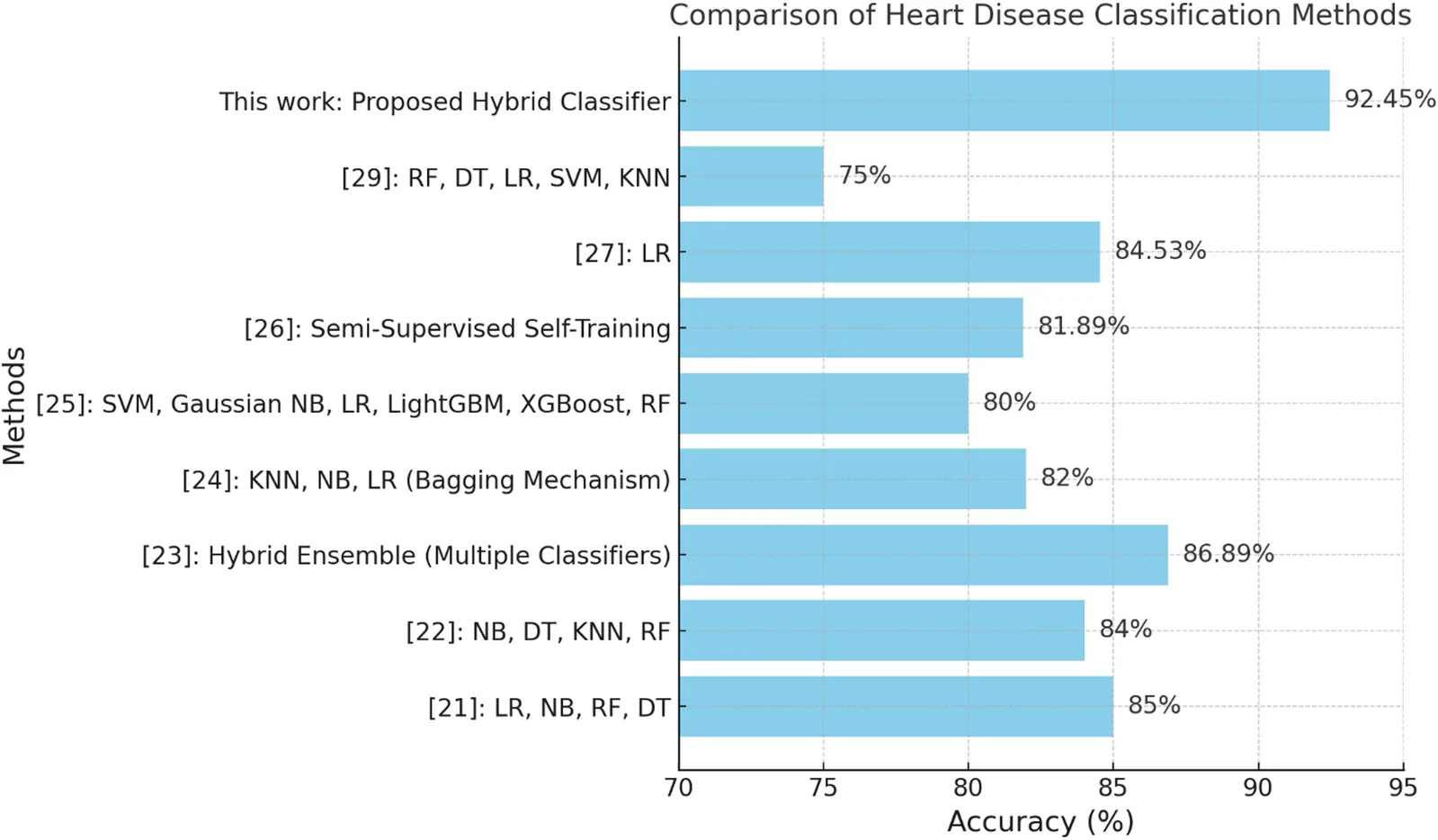
Comparison of heart disease classification methods with current work.

The recalibration mechanism in this model dynamically adjusts the importance of each feature based on its relevance to the prediction task. This helps the model filter out noise from less useful variables and concentrate on the most critical ones, improving both accuracy and specificity. In turn, this makes the system more adaptable to varied patient profiles, which is crucial for practical clinical decision-making and future healthcare applications Fig. 7.

The model’s performance becomes even more robust when ensemble learning is added, as it combines the strengths of multiple classifiers. While techniques like Random Forest, Extra Trees, and XGBoost are already proven across many domains, their effectiveness is significantly amplified when integrated with deep learning methods like autoencoders—as shown in this study. Ensemble learning helps reduce overfitting by blending predictions from multiple models, and it lowers the bias present in individual classifiers. This contributes to the hybrid model’s strong generalization ability, backed by its high sensitivity (93.2%) and specificity (91.4%).

When feature recalibration is combined with ensemble methods, it creates a powerful synergy: the ensemble improves generalizability, while recalibration ensures the most relevant features are appropriately weighted. This layered design gives the proposed hybrid classifier a distinct advantage over other ensemble models that don’t include deep feature extraction or self-adaptive feature recalibration.

Although past studies—like those reporting 86.89% accuracy for hybrid ensembles—have shown some improvement, they lack the multi-level integration used in this model. The superior performance of our hybrid approach shows the value of embedding advanced feature extraction and recalibration into the ensemble learning process, especially for complex, high-dimensional data like medical records.

Clinically, this model holds great potential for improving early detection and risk assessment in heart disease. Since heart disease is a global health concern, being able to reliably identify high-risk patients early means interventions can happen sooner, leading to better patient outcomes. High sensitivity ensures fewer missed cases, and high specificity helps avoid unnecessary tests—reducing both clinical risk and resource strain on healthcare systems.

However, the study does have some limitations. While the results are strong on the current dataset, the model needs validation on larger and more diverse datasets to ensure it works well across different populations. Risk factors vary by geography and ethnicity, so further testing in real-world clinical settings is essential. Additionally, future work should explore the use of temporal data—tracking changes in risk factors over time—to further improve predictions.

Finally, while effective, the model’s computational complexity—especially due to the use of autoencoders and ensemble methods—could pose a challenge in real-time clinical environments. Optimizing the system for speed and scalability will be a key focus of future research to make deployment in real-world settings more practical^24,31–36^.

## Limitations and future work

Despite the strong performance, this study has a few limitations. First, the dataset used is relatively small compared to large, multi-center clinical cohorts, which may affect how well the results generalize. Second, the model was tested only on a single structured dataset, without incorporating temporal trends or imaging data, which are common in real-world diagnostics. Third, the model has not yet been tested in real-time clinical settings, so its usability and integration into clinical workflows remain unverified. Future research should address these gaps to provide a more complete evaluation of the framework.

## Conclusions

The proposed hybrid framework greatly improves heart disease prediction by integrating deep feature learning, self-adaptive feature weighting, and confidence-based ensemble methods. Experimental results show that it outperforms both traditional and state-of-the-art models. With further testing on more diverse clinical datasets, this model holds strong promise for real-time use in diagnostic systems—supporting earlier detection and better patient outcomes.

Future work involving multi-center and cross-population datasets will be key to confirming its generalizability and supporting broader adoption in clinical practice. The proposed model demonstrates potential clinical relevance by accurately identifying high-risk patients based on non-invasive features. Such models can serve as supportive tools in primary care to prioritize further diagnostic testing.

The experiment findings prove that strict implementation of fold-wise learning protocol is much more effective in increasing model generalization and reliability. The suggested framework will offer a convenient and repeatable clinical implementation of a heart disease prediction system, which is applicable to real-world decision support systems in which heterogeneity of the data and the absence of bias are fundamental considerations.

## Data Availability

All data generated or analyzed during this study are included in this article.

## Declarations

Ethics declaration: Not applicable.
